# Five years of hospital based surveillance of influenza-like illness and influenza in a short-stay geriatric unit

**DOI:** 10.1186/1756-0500-7-99

**Published:** 2014-02-21

**Authors:** Corinne Régis, Nicolas Voirin, Vanessa Escuret, Byeul-A Kim, Olivier Robert, Bruno Lina, Brigitte Comte, Philippe Vanhems

**Affiliations:** 1 Université de Lyon, Université Lyon 1, CNRS UMR 5558, Laboratoire de Biométrie et Biologie Evolutive, 8 avenue Rockefeller, Lyon F-69373, France; 2 Hospices Civils de Lyon, Groupement hospitalier Edouard Herriot, Service d’Hygiène, Epidémiologie et Prévention, 5 place d’Arsonval, Lyon F-69437, France; 3 Hospices Civils de Lyon, Groupement hospitalier Est, Centre National de Référence de la Grippe, Région Sud, Laboratoire de Virologie, Centre de Microbiologie et de Pathologie Est, 59 boulevard Pinel, Bron F-69677, France; 4 Université de Lyon, Université Lyon 1, laboratoire EA4610, Laboratoire de Virologie et Pathologie Humaine, 7 rue Guillaume Paradin, Lyon cedex 08 F-69372, France; 5 Hospices Civils de Lyon, Service de Médecine Gériatrique, Groupement hospitalier Edouard Herriot, 5 place d’Arsonval, Lyon F-69437, France; 6 Hospices Civils de Lyon, Groupement hospitalier Edouard Herriot, Service de médecine Préventive, 5 place d’Arsonval, Lyon F-69437, France; 7 Service d’Hygiène, Epidémiologie et Prévention, Groupement hospitalier Edouard Herriot, Hospices Civils de Lyon, 5, place d’Arsonval, 69437, Lyon cedex 03, France

**Keywords:** Influenza, Hospital-acquired, Geriatrics, Surveillance

## Abstract

**Background:**

Data on influenza in the healthcare setting are often based on retrospective investigations of outbreaks and a few studies described influenza during several consecutive seasons.

The aim of the present work is to report data on influenza like illness (ILI) and influenza from 5-year prospective surveillance in a short-stay geriatrics unit.

**Findings:**

A short stay geriatrics unit underwent 5 years of ILI surveillance from November 2004 to March 2009, with the aim of describing ILI in a non-outbreak context. The study was proposed to patients who presented ILI, defined as fever >37.8°C or cough or sore throat. Among 1,353 admitted patients, 115 presented an ILI, and 34 had hospital-acquired ILI (HA-ILI). Influenza was confirmed in 23 patients, 13 of whom had been vaccinated. Overall attack rates were 2.78% and 0.02% for HA-ILI and HA-confirmed influenza respectively, during the 5 seasons.

**Conclusions:**

This 5-year surveillance study supports the notion that influenza infections are common in hospitals, mostly impacting the elderly hospitalized in short-stay units. It highlights the need for appropriate control measures to prevent HA-ILI in geriatric units and protect elderly patients.

## Findings

### Background

In healthcare settings, as in the community, influenza can cause severe illness or death in people at high risk, especially the elderly, since most mortality attributable to influenza occurs among people aged 65 years or older [1-5].

Most estimates of hospital-acquired influenza frequency are based on reports of outbreaks that occurred in long-term care units or nursing homes, where attack rate varied between 28% and 48% in 60 years-old persons [6]. On the one hand, these figures could have been overestimated because outbreaks with a large number of cases tend to be reported more often in the literature. On the other hand, influenza in elderly patients may be underestimated because clinical presentation can be altered by symptoms and treatments of pre-existing diseases [7,8]. A previous study explored the predictive values of influenza symptoms among hospitalized patients [9]. Despite a different study design and objective, it illustrated the need for additional data on influenza in the hospital context.

Data from long-term prospective investigations of influenza, especially hospital-acquired influenza, are lacking. The aim of the present study is to report data on influenza like illness (ILI) and influenza after 5-years prospective surveillance in a short-stay geriatric unit of a French university hospital.

### Methods

Prospective ILI surveillance was implemented between November and March from 2004 to 2007 in 32 wards of Edouard Herriot University Hospital in Lyon, France [10]. It was continued in 6 wards in 2007–2008 and 2008–2009. The geriatric unit, which is an 18-beds post-emergency hospitalization unit, participated continuously from 2004 to 2009, providing a unique opportunity to describe influenza and ILI during 5 consecutive years of geriatrics patient surveillance.

Study participation was proposed to each patient who presented ILI at admission or during hospitalization. ILI was defined as fever >37.8°C or cough or sore throat. Our definition converged towards that of the World Health Organization (fever >37.8°C with cough or sore throat), which was sensitive (98.4-100%) but not specific (12.9-21.5%) [11].

The following information was collected by oral interview and via medical charts: gender (male, female), age at admission, reason of admission (cardio respiratory disease without fever, infectious disease with fever, fall/confusion or other), vaccination against influenza during the ILI season (yes, no) and every season (yes, no), symptoms (fever > 37.8°C, cough and sore throat), complications (cardiac, respiratory) and death.

After the interview, a nasal swab was performed by a research assistant and influenza virus infection was confirmed by enzyme-linked immunosorbent assay and reverse transcription-polymerase chain reaction on viral cultures. Nasal swab samples were analyzed by the “Centre National de Référence de la grippe”. Respiratory syncytial virus (RSV), rhinovirus, parainfluenza virus and herpes simplex virus (HSV) were other viruses detected.

ILI was classified in 2 categories: community-associated ILI (CA-ILI), defined as patients who acquired ILI in the community or prior to hospitalization, and hospital-acquired ILI (HA-ILI), defined as patients with ILI acquired after admission. We considered ILI with onset occurring on the day of admission as CA-ILI but the time between admission and ILI onset was investigated.

To calculate attack rates, the number of monthly admissions to the unit was obtained from the hospital information system for the study period. Attack rates were calculated as the number of ILI or confirmed influenza among patients divided by the number of admissions at risk during the study period. In this calculation, we excluded, from the denominator, admissions for which patients presented with ILI or influenza (i.e. CA-ILI or CA-confirmed influenza) since we considered these patients to be no more at risk of influenza infection.

The data were analyzed by SPSS Statistics 17.0 (SPSS, Inc., Chicago, IL).

Informed consent was obtained from all patients. The study was approved by the French national bodies responsible for ethics and privacy, the “Commission Nationale de l’Informatique et des Libertés” (CNIL, http://www.cnil.fr/) and the “Comité de Protection des personnes” (http://www.cppsudest2.com/) of the hospital.

### Results

During the 5-year surveillance period, 1,353 patients were admitted in the unit and 115 patients presented ILI either at admission or during hospitalization (overall ILI burden of 8.5%). Table 1 reports on these 115 patients. No obvious difference was observed between CA-ILI and HA-ILI, except for a higher antibiotic use in CA-ILI. Among these 115 patients, 81 (70.4%) were CA-ILI and 34 (29.6%) were HA-ILI.

**Table 1:** Characteristics of patients with influenza like illness (ILI) from a geriatric unit from 2005-2009

**Characteristics**	**Community-acquired-ILI**	**Hospital-acquired-ILI**
	**n = 81**	**n = 34**
Gender: men/women	23/58	9/25
Median age (range, years)	88 (69–103)	88 (77–99)
Vaccination against influenza		
During the ILI season	45 (55.6%)	22 (64.7%)
Every season	42 (51.9%)	19 (55.9%)
Symptoms		
Fever > 37.8°C	47 (73.7%)*	23 (67.6%)
Cough	69 (85.2%)	30 (88.2%)
Sore throat	10 (12.3%)	5 (14.7%)
Length of stay (range, days)	6 (1–33)	8 (2–53)
Reason of admission		
Cardio respiratory disease without fever	39 (48.2%)	18 (52.9%)
Infectious disease with fever	34 (42.0%)	2 (5.9%)
Fall or confusion	16 (19.8%)	9 (26.5%)
Other	14 (17.3%)	10 (29.4%)
Treatment against ILI		
Antipyretics	49 (60.5%)	21 (61.8%)
Antibiotics	75 (92.6%)	23 (67.6%)
Antiviral treament	0 (0%)	0 (0%)
Lab results		
Influenza	17 (21.0%)	6 (17.6%)
Respiratory Syntytial Virus (RVS)	5 (6.2%)	0 (0%)
Influenza and RVS coinfection	1 (1.2%)	0 (0%)
Rhinovirus	3 (3.7%)	1 (2.9%)
Herpes Simplex Virus (HSV)	1 (1.2%)	0 (0%)
Parainfluenzae	1(1.2%)	0 (0%)
No detected virus	53 (65.4%)	27 (79.4%)
Complications		
Cardiac	3 (3.7%)	0 (0%)
Respiratory	6 (7.4%)	1 (2.9%)
Death	5 (6.2%)	4 (4.8%)

*ILI Percentages calculated for patients, n = 61; Data unknown for the other individuals.

Overall, among the 115 patients, no virus was found in 80 patients (69.6%) and at least one virus was detected in 35 patients (30.4%). Among these 35 patients, 23 (68.6%) presented a confirmed influenza and 11 (21.4%) had another respiratory virus: 5 had a respiratory syncytial virus (RSV), 4 had a rhinovirus, 1 has a herpes simplex virus (HSV) and 1 has a parainfluenza virus.

Among the 81 CA-ILI, 17 (22.2%) had confirmed influenza, 10 (12.3%) were infected with another virus (5 patients with a RSV, 3 with a rhinovirus, 1 with a HSV and 1 with a parainfluenza virus), 1 patient was coinfected with influenza and RSV, and no virus was identified in 53 (65.4%).

Among the 34 HA-ILI, influenza was confirmed in 6 (17.6%) patients and no virus was identified in 27 (79.4%). One patient was infected with rhinovirus.

**Figure 1:**
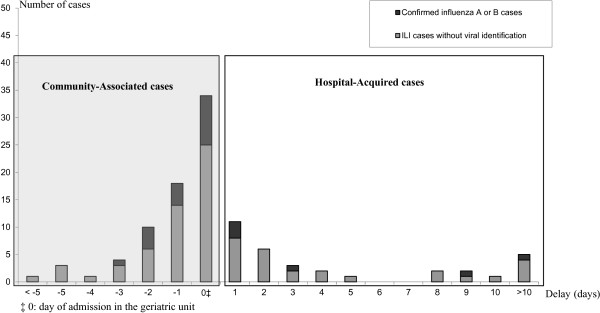
**Distribution of patients according to the duration between ILI onset and admission.** Distribution of number of patients with ILI according to the delay between onset of symptoms and admission (days) in geriatric unit during 5 years of surveillance at E. Herriot University hospital, Lyon (France).

Figure 1 reports the distribution of patients according to the duration between ILI onset and admission. Among the 34 HA-ILI, 12 (35.3%), 6 (17.6%), 3 (8.8%) and 13 (38.3%) presented ILI 24 hours, 48 hours, 72 hours and >72 hours after admission respectively. Among the 6 patients who acquired confirmed influenza in hospital, 3 were infected 24 hours after admission and the others 3, 9 and 27 days after admission.

Sixty-seven (58.3%) patients had been vaccinated against influenza (45 CA-ILI and 22 HA-ILI). Among these vaccinated patients, 13 (19.4%) declared confirmed influenza (i.e. vaccine failure) and 6 of them presented an influenza strain different from the vaccine strain (due to a mismatch in 2005). Among the 6 hospital-acquired influenza cases, 5 women had been vaccinated against influenza, 3 of them had an isolated strain different from the vaccine strain.

During ILI, 4 (3.5%) patients had cardiac complications and 6 (5.2%) had respiratory complications. Nine patients (7.8%) died from causes not directly attributable to ILI but 4 of them had HA-ILI.

Attack rates for HA-ILI regardless of viral identification were 3.7, 5.5, 1.8, 0.4 and 2.5 per 100 admissions from 2004–2005 to 2008–2009 respectively. Corresponding attack rates for HA-confirmed influenza were 1.4, 0.4, 0.3, 0 and 0 per 100 admissions.

### Discussion

Our findings indicate that HA-ILI occurred regularly and that influenza viruses are involved irregularly [5,6,12]. Our results revealed a number of infections by influenza viruses weaker than in outbreaks reports and that other viruses circulated in the hospitals, something that is rarely reported. The overall ILI attack rate in our geriatrics unit was 2.7%, highly comparable to the attack rate recorded by the “Groupes Régionaux d’Observation de la Grippe” (France), with an estimate of 2.64% for people aged ≥ 65 years during the same period [13]. For comparison, community epidemic curves for influenza from the “Groupes Régionaux d’Observation de la Grippe” are provided in Additional file 1.

In addition to these results on influenza frequency, this 5-year prospective study also gives an opportunity to discuss ILI and HA-ILI definitions and influenza vaccination.

The choice of ILI definition with broad criteria can be seen as not being adapted in our study but, with a more specific definition (e.g., fever >39°C with myalgia and a respiratory symptom), 23 (95.8%) patients with confirmed influenza would not have been included. This suggests that ILI definition is a key component of influenza surveillance in hospitals, especially since the elderly do not manifest influenza symptoms commonly found in the general population and co-morbidities alter influenza infection [7].

Our definition of HA-ILI can be also seen as not being adapted. We defined HA-ILI as ILI after admission but various definitions exist concerning nosocomial influenza. In our study 50% of hospital-acquired confirmed influenza declared their symptoms 24 hours after admission. For influenza, it has been suggested that 30% of infected persons may develop symptoms within one day, and 80% within two days, after infection, with an average incubation period of 1.9 days [14,15]. We, therefore, consider that HA-ILI may be the result of infection by the virus in hospital within the first day but also within the second day of admission. Altogether, the definition of nosocomial ILI or nosocomial influenza remains an open issue.

Several studies have demonstrated the effectiveness of influenza vaccination in seniors [16], although potential biases could represent certain limitation [17,18] and age-related immunodeficiency could impair vaccine efficacy [19]. In our investigation, more than 50% of individuals with confirmed influenza had been vaccinated. This high rate of vaccine failure could be related to low efficacy of influenza vaccination in the elderly, as reported previously [20]. However, we noticed that 6 individuals had been infected with a strain different from the vaccine strain, and that 68% of ILI occurred in February or March, more than 3 months after the influenza vaccination campaign. In France, influenza vaccination is recommended for people aged 65 years or older but is not mandatory. We suppose it may explain the low influenza vaccine coverage in this population.

The strength of our study is its prospective design and 5-year duration of surveillance. However, selection bias towards more severe cases could have been a limitation since hospitalized patients in a university centre could be more affected by underlying diseases than in other geriatric institutions, such as long-term care facilities.

Influenza is difficult to detect in hospital as its diagnosis is based on nonspecific symptoms. Treatment may mask the symptoms and the diagnosis of suspected nosocomial pulmonary infection is mainly attributed to bacterial rather than viral identification, as may be attested by the frequent use of antibiotherapy in our study. Although viruses are supposed to be a major cause of nosocomial infection [21,22], a lack of data prevents us from estimating the proportions of viral infections among nosocomial infections. A proportion of 5% has been suggested, but this value is probably underestimated. The low frequency of viral nosocomial infections compared to bacterial infections may be explained by more difficult detection due to longer and varying incubation times, a significant proportion of asymptomatic infections, or a low rate of antiviral resistance. Not surprisingly, our study reported low attack rates and, furthermore, a few other respiratory viruses were detected.

### Conclusion

Although hospital-acquired influenza detection and estimates are imprecise, nosocomial influenza is a reality with a potentially significant impact in the elderly hospitalized in short-stay units. While the beneficial effects of vaccinating the elderly are being debated, it appears important to protect this vulnerable population by decreasing its exposure to the virus, by vaccinating healthcare workers and by applying infection control measures including hand washing, disinfection and use of masks [23].

## Competing interests

Professor Bruno Lina has received travel grants from Roche; research grants from Roche and sanofi Pasteur, and consulting fees from Roche, GlaxoSmith-Klein, Biocryst, Merck, and sanofi pasteur. The other authors have no conflicts of interest.

## Authors' contributions

CR contributed to study design, data acquisition, analysis and interpretation, and drafted the manuscript. NV contributed to data analysis and interpretation, and drafted the manuscript. PV, BL and BC contributed to study design and manuscript revision. VE, BAK and OR contributed to data interpretation and manuscript revision. All authors read and approved the final manuscript.

## Supplementary Material

Additional file 1 **Epidemic curves of estimation of medical consultation for acute respiratory infection (IRA) by age groups (years) in France (per 100,000 inhabitants) for each season of our study.** Adapted from Réseau des GROG [http://www.grog.org]. Click here for file
