# Supplementary material for: Five years of hospital based surveillance of influenza-like illness and influenza in a short-stay geriatric unit
Source: BMC Res Notes. 2014 Feb 21;7:99. doi: 10.1186/1756-0500-7-99 (PMC3943500; doi:10.1186/1756-0500-7-99)

Additional file 1

Epidemic curves of estimation of medical consultation for acute respiratory infection (IRA) by age groups (years) in France (per 100,000 inhabitants) for each season of our study

Adapted from Réseau des GROG [http://www.grog.org]

2004/2005 season

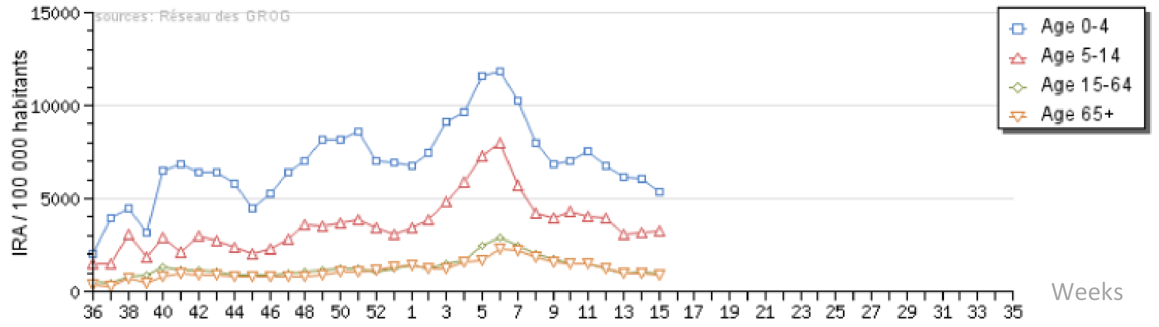

2005/2006 season

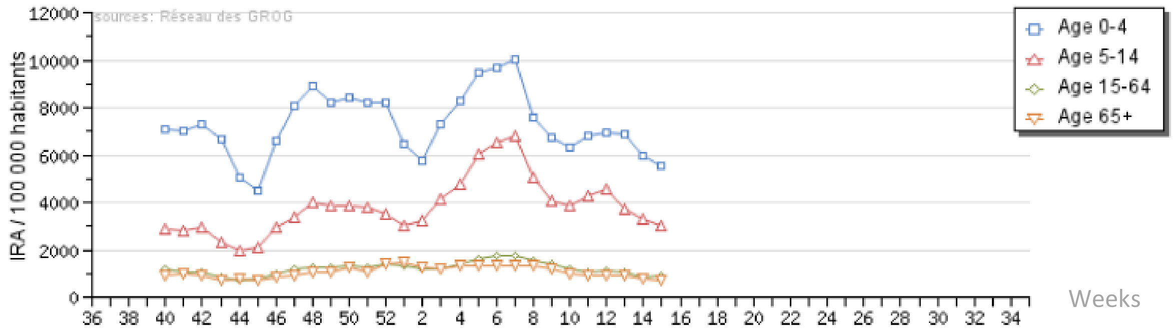

2006/2007 season

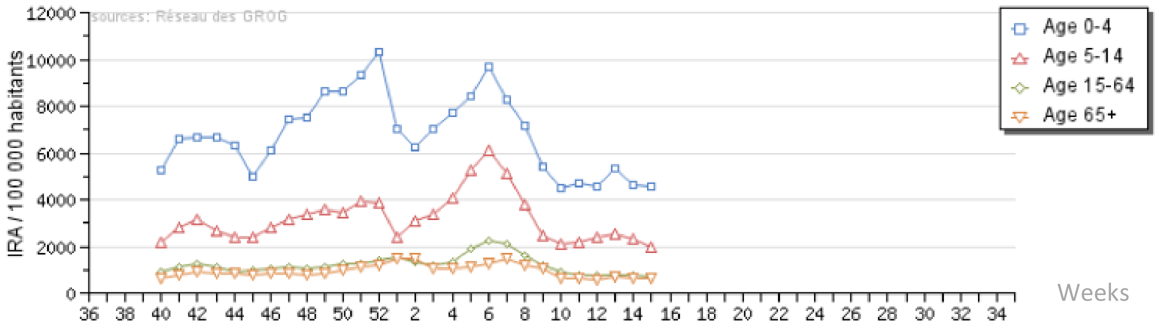

2007/2008 season

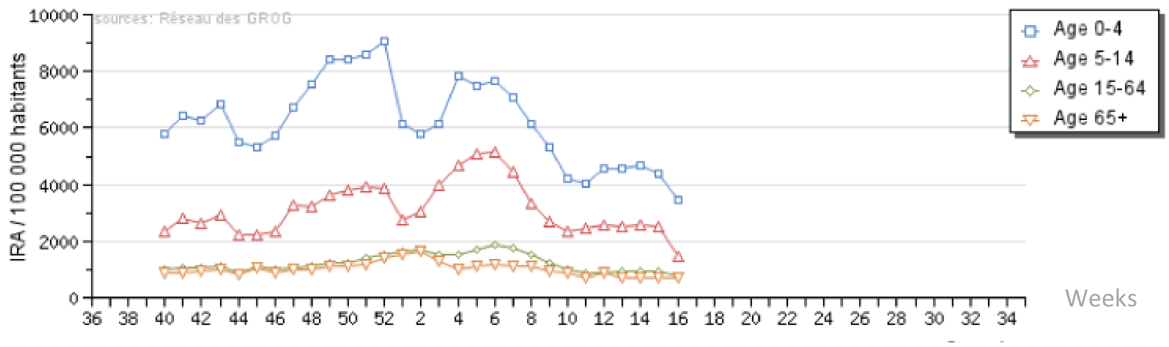

2008/2009 season

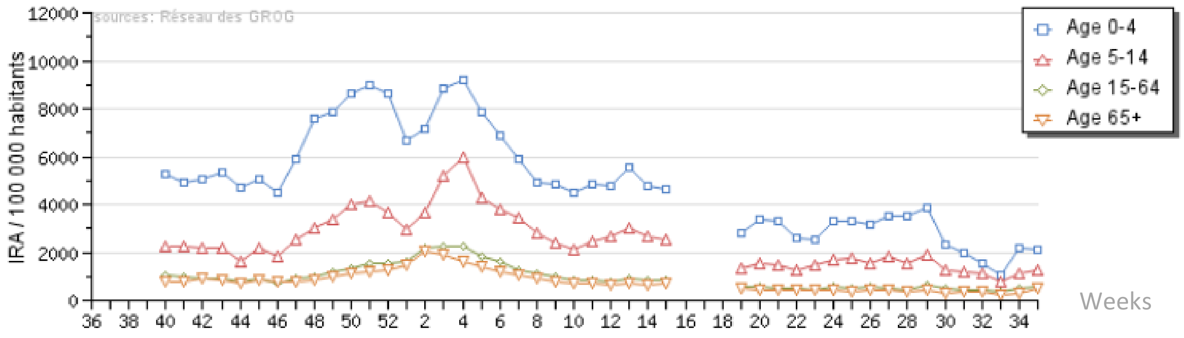

Supplement: Additional file 1 — Epidemic curves of estimation of medical consultation for acute respiratory infection (IRA) by age groups (years) in France (per 100,000 inhabitants) for each season of our study. Adapted from Réseau des GROG [http://www.grog.org]. [file 1756-0500-7-99-S1.pdf]
